# Supplementary material for: Regulatory Effects Mediated by Enteromorpha prolifera Polysaccharide and Its Zn(II) Complex on Hypoglycemic Activity in High-Sugar High-Fat Diet-Fed Mice
Source: Foods. 2023 Jul 27;12(15):2854. doi: 10.3390/foods12152854 (PMC10417851; doi:10.3390/foods12152854)
Supplement: Supplementary file 1 [file foods-12-02854-s001.zip › foods-2461559-supplementary.pdf]

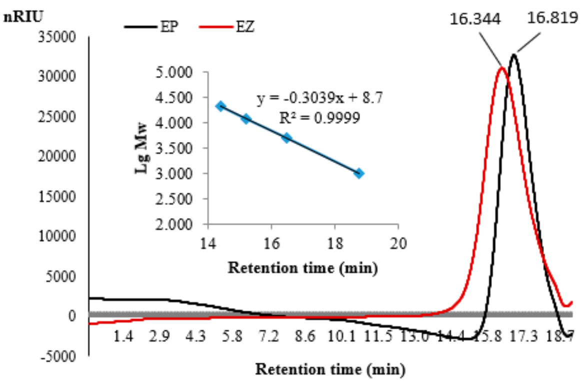

Fig.S1 HPGPC chromatogram of EP and EZ.

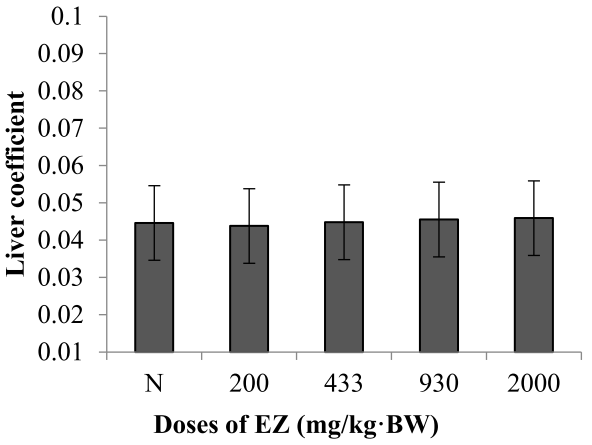

(a)

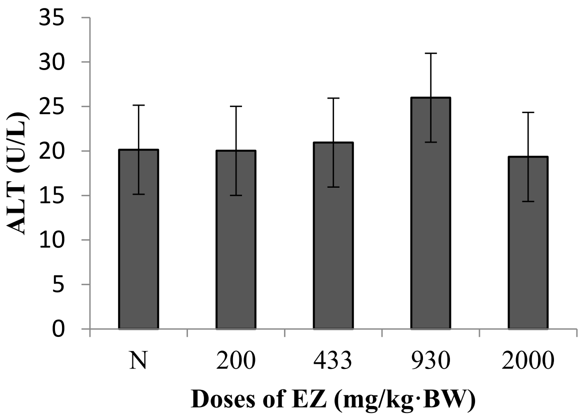

(b)

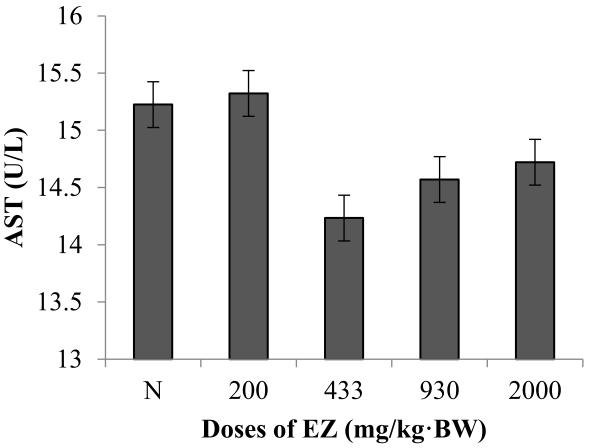

(c)

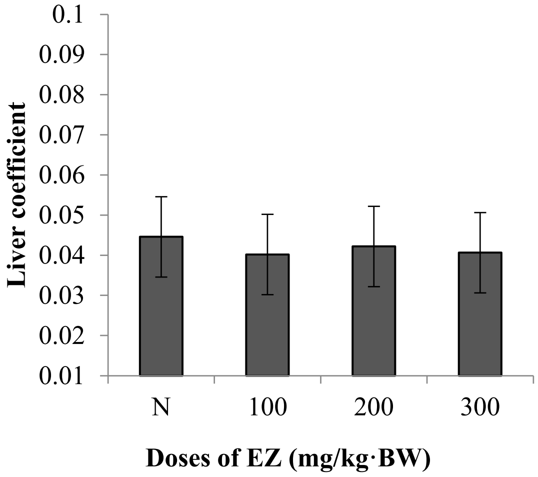

(d)

Fig.S2 Effect of EZ in different doses on liver coefficient, ALT and AST levels in ICR mice (n=8). N, normal group. (a)Liver coefficient in acute toxicological dose group; (b)ALT in acute toxicological group; (c) AST in acute toxicological group; (d) Liver coefficient in subacute toxicological group. $p>0.05$ for all data vs the normal control group.

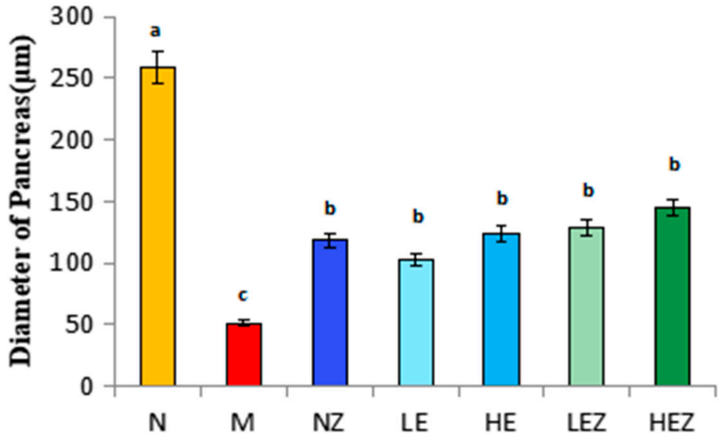

FigS3. Effects of EZ on size of pancreas in high-sugar and high-fat diet-fed mice (n=8) and compared at maximum horizontal diameter of pancreas.

Table S1. Primer sequences for qRT-PCR.

Gene	Forward	Reverse
IRS-2	CACAATTCCAAGCGCCACAA	CATCACCTCCTCCCAGGGTA
PI3K	CCAGTAGGCAACCGTGAAGA	TGTTCCTTCGAAAGTCTTGGAC
AKT	CTGTGTGTGCTGGGGGTC	CTTCCCACAGTTCCTCTGGC
GLUT2	TGTTGGGGCCATCAACATGA	TGCCAATCATCCCGGTTAGG
β-actin	TGTCCACCTTCCAGCAGATGT	AGCTCAGTAACAGTCCGCCTAGA
